# Cause-Effect Relationships between Painful TMD and Postural and Functional Changes in the Musculoskeletal System: A Preliminary Report

**DOI:** 10.1155/2022/1429932

**Published:** 2022-02-28

**Authors:** Adam Andrzej Garstka, Monika Brzózka, Aleksandra Bitenc-Jasiejko, Roman Ardan, Helena Gronwald, Piotr Skomro, Danuta Lietz-Kijak

**Affiliations:** ^1^Department of Propaedeutic Physical Diagnostics and Dental Physiotherapy, Faculty of Medicine and Dentistry, Pomeranian Medical University, Szczecin, Poland; ^2^Doctoral Study, Department of Propaedeutic Physical Diagnostics and Dental Physiotherapy, Faculty of Medicine and Dentistry, Pomeranian Medical University, Szczecin, Poland; ^3^Department of Econometrics, Faculty of Economic Sciences, Koszalin University of Technology, Koszalin, Poland

## Abstract

**Introduction:**

Temporomandibular disease (TMD) is a general term including a group of conditions that cause pain and dysfunction in the masticatory muscles, the temporomandibular joint (TMJ), and their related structures. The painful forms of these dysfunctions have become an increasing phenomenon among dental patients. A number of scientific publications indicated the relationship between the presence of postural dysfunctions and functional disorders of the masticatory system in humans. Nevertheless, dental procedures still very rarely include comprehensive diagnostics and procedures aimed at the normalization of the locomotor system related to TMD. Scientific literature usually refers to and describes the coexistence of postural disorders in patients with TMD in the context of anatomical connections, the so-called biokinematic chains, indicating specific types of postures that correlate with different positions of the mandible and/or teeth.

**Objective:**

The aim of the study was to investigate the effect of painless positioning of the mandibular head in the articular fossa on postural and functional changes in the musculoskeletal system.

**Materials and Methods:**

The study was conducted on a group of 30 randomly selected patients who reported to the Department of Propaedeutic, Physical Diagnostics and Dental Physiotherapy of the Pomeranian Medical University in Szczecin (Poland). Before the examination, the dentists and the physiotherapist were calibrated by an examiner who had previously been calibrated and had three years of experience in the management of patients with TMD. Training of the appropriate palpation strength was performed, and then the results were discussed. In the study group, painful disorders in the temporomandibular joint with an abnormal position of the mandibular head in the articular fossa and individual posture defects were found. The patients complained of pain in the area of the TMJ, episodes of locked joints, and difficulty biting. None of them was treated for these disorders, previously rehabilitated or participated in any body posture examination. The patients were examined by an interdisciplinary team who also performed a preliminary test. The inclusion criterion for the study group was the presence of TMD symptoms in the past. Myofascial pain was diagnosed on the basis of diagnostic criteria for temporomandibular disorders (RDC/TMD Ia and Ib). On the other hand, the displacement of the articular disc was diagnosed on the basis of the diagnostic criteria of temporomandibular disorders (RDC/TMD IIa)—displacement of the articular disc without reduction. At the same time, the body posture was assessed by inspection and using computer techniques while standing and during motion. The examinations were repeated after positioning the mandibular heads in the articular fossa and stabilizing the condylar process using a temporary silicone occlusal splint. Since there is no DC/TMD protocol in Polish to date, RDC/TMD was used in the study.

**Results:**

Initial pilot studies and the authors' observations indicated that the positioning of the mandibular heads in the articular pits and stabilization of the condylar process by providing the oral cavity with a temporary, silicone occlusive splint significantly influenced the posture of the examined patients, both while standing and during locomotion. This correlation also applies to the corrective effect on the foot architecture during standing and patient gait.

**Conclusions:**

Diagnostic and therapeutic management in the course of TMD should be holistic. Nevertheless, the observed changes are often varied and largely dependent on individual posture defects, which is an important postulate for further research on a larger study group.

## 1. Introduction

TMDs such as occlusion disorders, displacement of the articular disc, increased tension of the masticatory muscles, bruxism, and acoustic phenomena in the temporomandibular joint affect an increasing number of patients [1]. Often, these problems coexist with other musculoskeletal disorders such as protraction head, abnormal position of the pelvis, and the angle of lumbar lordosis, within the knee joints or the architecture of the feet [2, 3]. Patients with any type of TMD may have several symptoms otological symptoms such as tinnitus, ear fullness, ear pain, hearing loss, hyperacusis, and vertigo, which may be due to the anatomical proximity between the temporomandibular joint, muscles innervated by the trigeminal nerve, and ear structures [4]. The relationship between dental occlusion and ophthalmology attracts less attention, and yet, it is also important [5, 6].

TMJ connecting the mandible with the temporal bone, with articular surfaces between which the articular disc is located, is one of the most important parts of the masticatory and speech organs. This joint is made of hard and soft tissues and is considered to be one of the most complicated joints in the human body. Its proper operation is responsible for the basic, physiological activities such as speaking, swallowing, and eating [7–9]. The dysfunctions within it are manifested through crackling, pain, headache, and mobility disorders [10–15]. Under normal conditions, without disturbances in the TMJ mandibular, the patient does not feel pain, discomfort, acoustic phenomena, locks, and problems in the mobility of the mandible. Pain is a problem with mandibular abduction, temporary or permanent locks, and acoustic phenomena such as crackles or clicks. In the case of disorders of muscle origin, the intra-articular situation is correct, but there is a difference in speed and the range of individual movements of the mandible, which are most often accelerated, i.e., acceleration compared to healthy patients [16–20]. Similar conclusions were formulated by Yokoyama et al., additionally pointing out that an increase of speed caused acoustic phenomena having a tendency to be heard in a closed position of the condylar process [21]. The purpose of the preliminary test is to check for the existence of a musculofascial, according to the concept of Anatomy Trains, and examine whether the temporomandibular disorder can linearly affect distant tissues [22]. According to the tensegration theory, the damaging stimulus caused by excessive voltage, it is transmitted linearly in the human body [23–26]. For this reason, ailment pain and mobility limitations may appear in a place distant from the primary stimulus. According to the concept of Anatomy Trains, there is a network of visual connections in the human body [27]. TMJ through the myofascial and ligamentous connections with the cervical segment forms a functional relationship. Therefore, patients with disorders in the TMJ are people who automatically position their heads in protection, which results in deepening cervical lordosis and the presence of pelvic dysfunction [28–34]. Clinical studies have shown a direct link between the abnormal setting of the shoulder and pelvic girdles to the incorrect position of the mandible [35]. Relationships between disorders in the temporomandibular joint and existing dysfunctions in the cervical spine have been confirmed by clinical tests [36–39]. The presented pattern of a posture of a human body with disturbances present in the TMJ through anatomical tapes could linearly influence other human body segments, e.g., through the trapezius and levator scapula deepen the lumbar lordosis, which, by way of compensation, is involved in increased hip flexion and pelvic anterior tilt, which directly affects the curvature of the spine. Studies have been carried out describing the impact of disorders within the pelvis on the TMJ and the relationships in the reverse order [40–43]. A properly constructed and functional pelvis with undisturbed sacroiliac joints is found to be a key region in static balance [44]. Disorders of the pelvis, its components and sacroiliac joints, cause a static imbalance manifested as disorders of other remote parts of the body, including the TMD [44]. The sacroiliac joint is a gliding joint, formed by the iliac and sacrum bone, strengthened by the interosseous sacroiliac ligaments, abdominal sacroiliac ligaments, and dorsal sacroiliac ligaments. Iliolumbar, sacrospinous, and sacrotuberous ligaments indirectly strengthen the sacroiliac joint. The range of motion in the sacroiliac joint is minimal. Therefore, any disorder of this joint and its connections with the structures mentioned above leads to dysfunction [45]. Additionally, the feet and toes play a key role in static balance and proper gait.

These relevant reports indicate the need to combine dental diagnostic procedures of TMD with a posture examination. In particular, taking into account the anatomical and, above all, biomechanical complexity of the locomotor system. Given the above, it is an important task of interdisciplinary teams dealing with dental therapy to conduct a detailed dental diagnostic in almost every procedure combined with a posture examination (while standing and in motion-free walking).

## 2. Objectives

The aim of the study was to investigate the effect of painless positioning of the mandibular head in the articular fossa on postural and functional changes in the musculoskeletal system.

### 2.1. Materials and Methods

The study was conducted on a group of 30 randomly selected patients who reported to the Department of Propaedeutic, Physical Diagnostics and Dental Physiotherapy of the Pomeranian Medical University in Szczecin (Poland). Before the examination, the dentists and the physiotherapist were calibrated by an examiner who had previously been calibrated and had three years of experience in the management of patients with TMD. Training of the appropriate palpation strength was performed, and then the results were discussed. The RDC/TMD protocol was modified with acoustic phenomena because the vast majority of patients reporting to the clinic report the presence of sounds during the abduction and adduction movements of the mandible. The inclusion criterion for the study group was the presence of TMD symptoms in the past. The study population suffered from TMD with improper positioning of the mandibular head and individual postural defects. The subjects had no prior diagnosis of TMD. The study population did not experience systematic diseases, including craniofacial, spine, and pelvic injuries, nor neurological and psychiatric disorders. The patients complained of TMJ soreness, episodes of locked joints, and difficulty biting. None of them was treated for these disorders, previously rehabilitated or participated in any body posture examination. Individuals undergoing a regular drug therapy and those with mental illness, coagulopathy, diabetes, and chronic infections were excluded from the study. The subjects were not addicted to nicotine, alcohol, or illegal substances.

The research protocol was approved by the Bioethics Committee of the Pomeranian Medical University in Szczecin (Poland) (KB-0012/126/17). The study complied with the ethical standards. All participants signed a written informed consent form and were instructed on the technique and course of the research. The patients were examined by an interdisciplinary group who also performed a preliminary test. The subjects completed a personal questionnaire. A full general and dental medical history was collected, with particular emphasis on TMD.

### 2.2. Dental Examination Methodology

The dental analysis was conducted on the basis of the modified Research Diagnostic Criteria for Temporomandibular Disorders (RDC/TMD Ia, Ib, and IIa). The analysis also included acoustic phenomena within the joint and heard during jaw movements. The phenomena were evaluated using the following criteria: 0-no sound, 1-click, 2-cracking, pain during movements (0-no pain; 1-the presence of pain), and pain on palpation of the posterior and lateral regions of the TMJ (0-no pain; 1-the presence of pain). Palpation of the lateral and posterior parts of the joints was conducted in order to diagnose painful areas. Acoustic phenomena were examined using a stethoscope, with the chest piece applied to the lateral and posterior surfaces of the TMJ.

The examination of TMD was performed in a supine position, as it provides the most accurate sightline into the oral cavity and the best ergonomics of the examination. The examination started with correct positioning of the head on the headrest, the Camper line was perpendicular to the ground, the orbital plane was parallel to the ground, and the head was set at the height of the knees. Maintaining the supine position allowed the examination to be repeated in the same patient, as well as on the entire group of subjects.

Direct inspection of the facial features allowed for a visual assessment of the facial symmetry, and for checking the absence or presence of lesions or swelling. The correct sequence of the actions was important for the repetition and reliability of the results. The examinations started at the shoulder girdle muscle, then the examiners moved toward the temporal muscles. Muscle palpation was performed in order to detect trigger points and to assess tension in the temporal muscles, medial pterygoid, lateral pterygoid, masseter, suprahyoid, deltoid, sternocleidomastoid, and rhomboid muscles.

**Figure 1:**
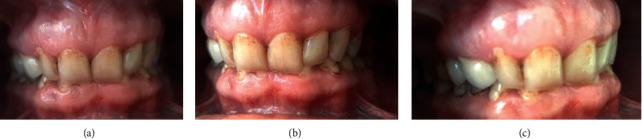
(a–c) An intraoral examination in order to determine the condition of the teeth and the presence of occlusion defects and to examine the periodontal tissues. (a) The assessment of the patient's occlusion-overbite. (b) The bite assessment according to Angle's classification and canine classification-left side. (c) The bite assessment according to Angle's classification and canine classification-right side.

An intraoral examination was also performed in order to determine the condition of the teeth (DMF) and the presence of occlusion defects and to examine the periodontal tissues (CPITN) (Figures 1(a)–1(c)).

After the dental examination, the patient was referred to a physiotherapist who performed the postural examination.

### 2.3. Methodology of the Physiotherapeutic Examination

**Figure 2:**
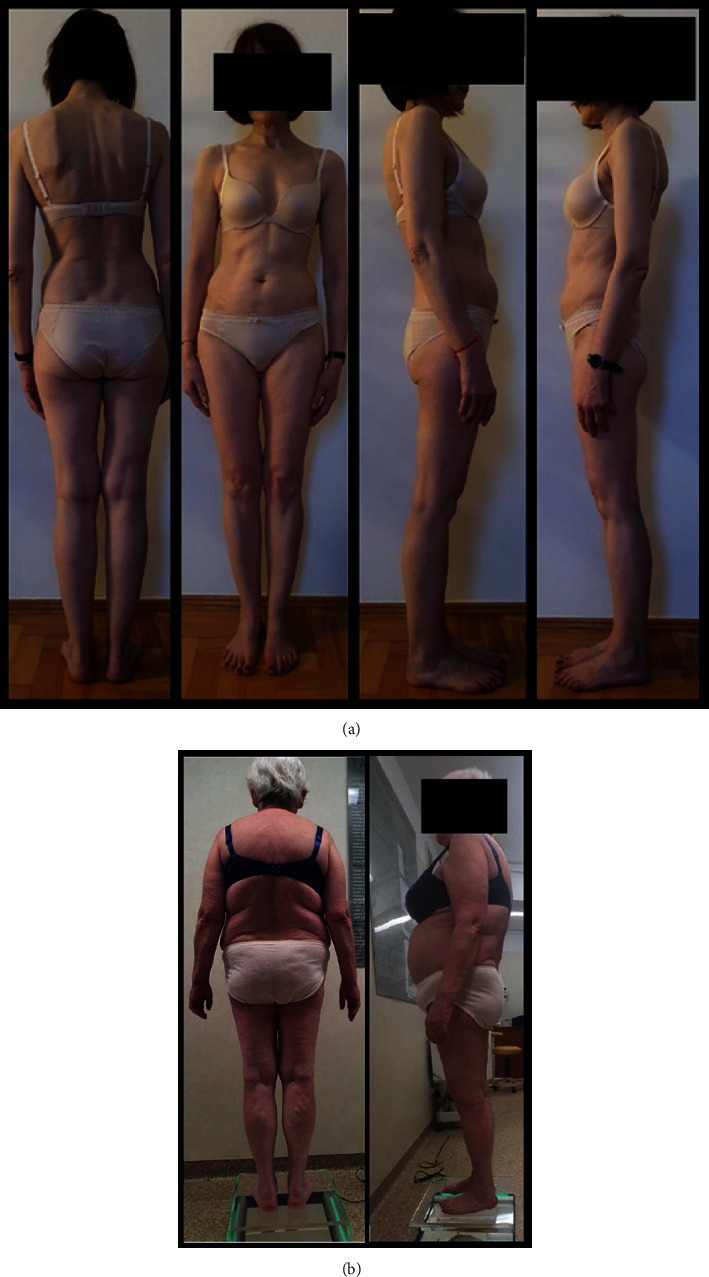
(a) A visual examination of an exemplary patient. (b) A visual examination of an exemplary patient.

Each patient underwent the following subjective and objective physiotherapeutic examinations: visual assessment of the posture, positioning asymmetry in the patient's body, and comparisons of individual body sequences in three planes: frontal, sagittal, and transverse performed using a podoscope, goniometer, and ruler (Figures 2(a) and 2(b)). Next, functional tests of the sacroiliac joints were carried out (asymmetry of the lower limbs length and excessive joint mobility were excluded in each patient-spring test II). The iliac spine test and the standing flexion test were carried out. A pedobarographic examination was also performed along with the assessment of standing and gait, as well as pelvic function testing using the Wiva Science sensor, equipped with a sensomotor magnetometer system and a gyroscope. The results of the pelvic range of motion were obtained during walking in 3 planes: forward/backward inclination, lowering and lifting the iliac alae, and rotation.

The subjects had a negative history of spine and temporomandibular joint injuries; they also had not undergone any surgery and did not have scoliosis or other postural defects diagnosed in childhood.

**Figure 3:**
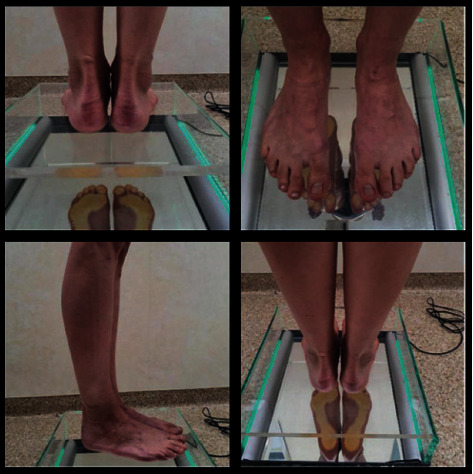
A visual examination of an exemplary patient using a podoscope.

The following structures and body segments were assessed:

- Head position and head-to-shoulder ratio
- Position of the lower jaw and its relation to the scalp
- Position and protrusion of the arms, their relationship to each other, and the lumbar spine
- Position of the shoulder blades, their relationship to each other, and the spine
- The ratio of the cervical segment to the thoracic and lumbar segment
- The ratio of the thoracic segment to the lumbar segment
- Waist indentation line
- Navel position
- Posterior superior and inferior iliac spine and their relationship
- The pelvis and its position relative to the upper and lower segment of the body
- Iliac alae and their relationship
- Symmetry of the buttock line
- Popliteal pits line
- Position of the patella and the knee to medial ankle ratio
- Feet: tarsus, metatarsus, toes, and vault (Figure 3)

**Figure 4:**
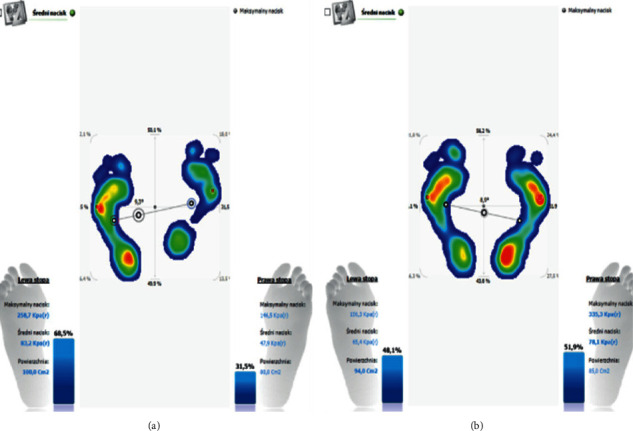
(a) Distribution of pressure in the anterior/posterior and lateral view and the angle of the relation of the feet before repositioning the mandibular head in the articular fossa and stabilizing the position of the condylar process with a temporary silicone occlusive splint. (b) Distribution of pressure in the anterior/posterior and lateral view and the angle of the relation of the feet after repositioning the mandibular head in the articular fossa and stabilizing the position of the condylar process with a temporary silicone occlusive splint.

**Figure 5:**
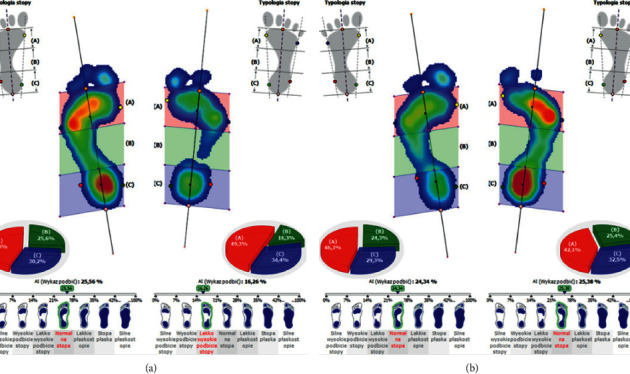
(a) The results of the AI test before repositioning the mandibular head in the articular fossa and stabilizing the position of the condylar process with a temporary silicone occlusal splint, in an exemplary patient. (b) The results of the AI test after repositioning the mandibular head in the articular fossa and stabilizing the position of the condylar process with a temporary silicone occlusal splint, in an exemplary patient.

**Figure 6:**
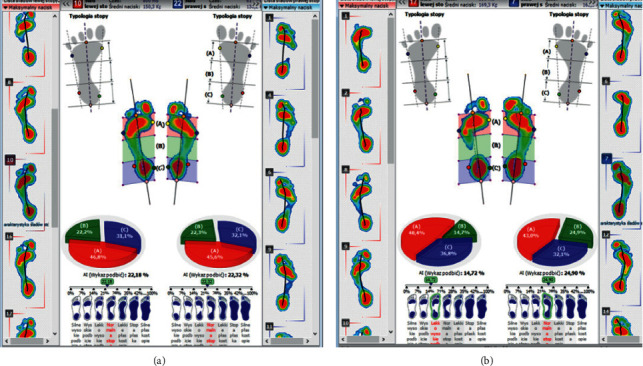
(a) The results of the AI test before repositioning the mandibular head in the articular fossa and stabilizing the position of the condylar process with a temporary silicone occlusal splint, in an exemplary patient, during walking. (b) The results of the AI test after repositioning the mandibular head in the articular fossa and stabilizing the position of the condylar process with a temporary silicone occlusal splint, in an exemplary patient, during walking.

During the podoscopic examination, the following anthropometric measurements were taken: arch of the foot, the position of the tarsus, fingers, knees, etc. During the examination, functional tests of the sacroiliac joints were performed along with the evaluation of the length of the lower limbs (joint mobility-spring test II). A differentiation test, also known as the iliac spine test, was carried out in order to assess the function of the sacroiliac joints. Postural and functional diagnostics were conducted using computer methods. A pedobarographic test (EPS R1 pedobarograph, BIOMECH Studio v2 software) was conducted to evaluate the arch of the foot and the distribution of pressure on the feet. The examination was performed during standing and walking (motion), which allowed for the assessment of the relationship between the feet based on the deviation angle COP (Figures 4(a) and 4(b)) and the AI (Arch Index; Figures 5(a), 5(b), 6(a), and 6(b)).

**Figure 7:**
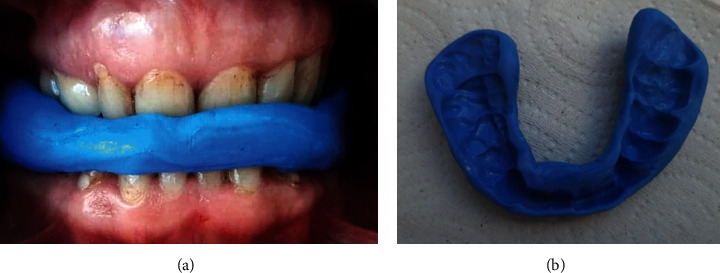
(a, b) Stabilization of the mandibular head position in the center using a temporary silicone occlusal splint (CyberTech silicone putty; DE Healthcare Products Gillingham ME8 OSB U.K.).

The described physiotherapeutic examination was performed before and after changing the position of the mandibular head in the articular fossa, which was stabilized by the correction of the stomatognathic system using a temporary silicone occlusal splint (Figures 7(a) and 7(b)).

**Figure 8:**
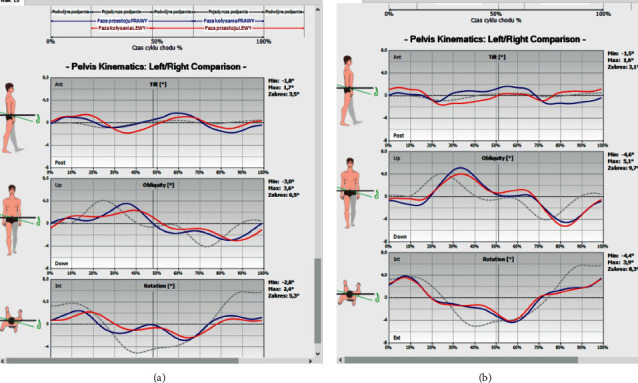
(a) The result of the pelvic mobility test and angle of lordosis (while standing) before repositioning the mandibular head in the articular fossa and stabilizing the condylar process with a temporary silicone occlusive splint in an exemplary patient. (b) The result of the pelvic mobility test and angle of lordosis (while standing) after repositioning the mandibular head in the articular fossa and stabilizing the condylar process with a temporary silicone occlusive splint in an exemplary patient.

The functional assessment of the pelvic complex was performed using computer techniques of postural diagnostics and functional evaluation (Wiva Science biokinetic sensor, BIOMECH Studio software) (Figures 8(a) and 8(b)). The angle of lordosis, measured while standing, and the range of motion were determined in three planes during walking:

- Frontal plane: the range of motion assessed by lifting/lowering the iliac alae
- Sagittal plane: the range of motion of the pelvis tilted forward and backward
- Transverse plane: the range of motion of the pelvis relative to the lumbar region and rotation associated with alternating movements of the lower limbs

The next stage of the diagnosis included a dental intervention by placing the head of the mandible in the center, the elimination of the acoustic phenomena in all patients during the adduction and abduction movements of the mandible, and stabilization of this position by means of a temporary silicone occlusal splint. Then, postural tests using computer techniques were carried out again while standing and during motion.

After the examinations, the patients were treated for a period of 1 to 3 months with a bimangled reposition brace and were subjected to intensive physiotherapy. After this time, patients were presented with orthodontic, prosthetic, and conservative treatment plans depending on their needs.

## 3. Results

The paired *t*-test was used in statistical analysis to compare the characteristics before and after therapy. The normality of the distribution of quantitative features was checked using the Shapiro–Wilk test. The results were considered statistically significant at *p* < 0.05. The R statistical package was used for the calculations.

The use of a temporary silicone occlusal splint to change the position and the correct positioning of the mandibular head in the articular fossa had a positive effect on the acoustic phenomena within the temporomandibular joint, eliminating the sounds in 100% of patients. The results of measurements of acoustic abnormalities were digitized as follows:

- 0: absence of sound
- 1: click
- 2: crackling

**Figure 9:**
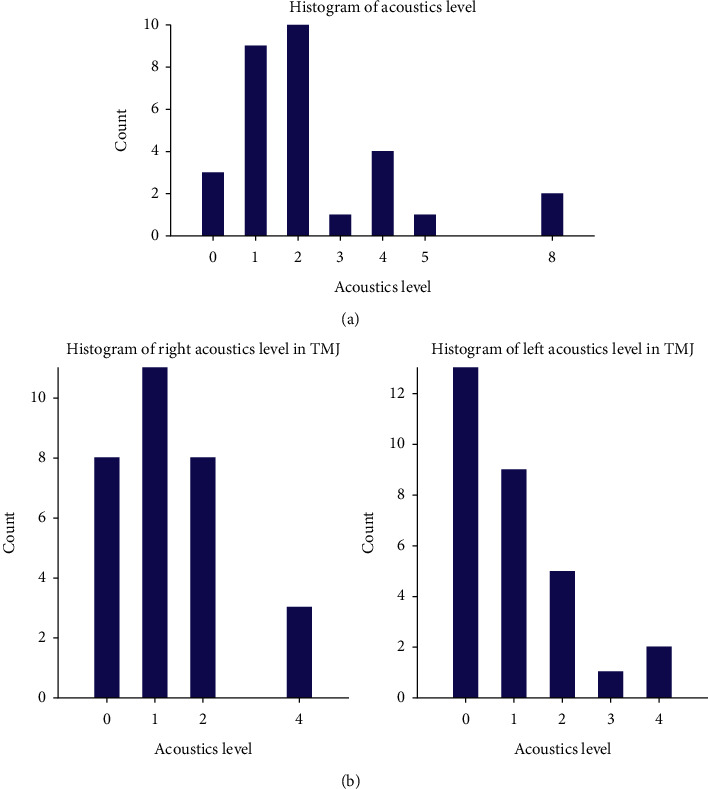
(a) Histogram of general acoustic abnormalities. (b) Histogram of acoustic abnormalities with division into the left and right TMJ.

The general level of acoustic abnormalities was calculated as the sum of the results of four measurements of mandibular movements. The distribution of acoustic abnormalities is presented in the histogram in Figure 9(a). The histogram of acoustic abnormalities with division into the left and right sides is shown in Figure 9(b).

Next, the presence of TMJ pain was examined during the abduction and adduction of the mandible. After stabilizing the position of the mandibular head in the articular fossa using a temporary silicone occlusal splint, TMJ pain was absent in 100% of the patients. Then, the pain in the lateral and posterior part of the temporomandibular joint was evaluated twice: in a preliminary examination and after stabilizing the position of the mandibular head in the articular fossa using a temporary silicone occlusal splint. The functional tests were performed to assess the locks of the sacroiliac joints, with the presence of locks coded with number 1 and the physiological condition (no locks) coded with number 0. Locks of both joints were reported in 13 patients, locks of the right joint occurred in 5 subjects, locks of the left joint were observed in 11 individuals, and one patient had no locks at all. The general level of locks was accepted as the sum of these two variables (it was 2 in 13 patients, 1 in 16 patients, and 0 in 1 patient). The correlation between the level of TMJ acoustic abnormalities and the sacroiliac joint locks was also analyzed (Table 1).

**Figure 10:**
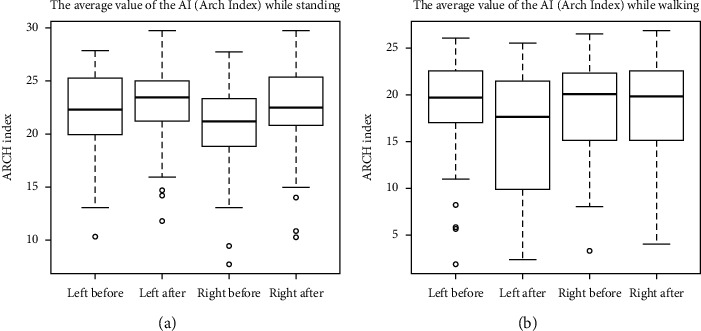
(a) Distribution of the AI before and after stabilizing the mandible in a new position using a temporary silicone occlusal splint; the examination was performed in the standing position. (b) Distribution of the AI before and after stabilizing the mandible with a temporary silicone occlusal splint; the examination was performed during walking.

There is a statistically significant positive correlation between the level of TMJ acoustic abnormalities and the sacroiliac joint locks, both on one side and in the general level of locks. Statistical analysis of changes in the arch of the foot showed that after removing outliers, there are significant differences in the mean values of the AI, except for the result obtained for the right feet, during walking (Table 2). The distribution of the results of the AI before and after stabilizing the mandibular head in the articular fossa using a temporary silicone occlusal splint is shown in Figures 10(a) and 10(b).

**Figure 11:**
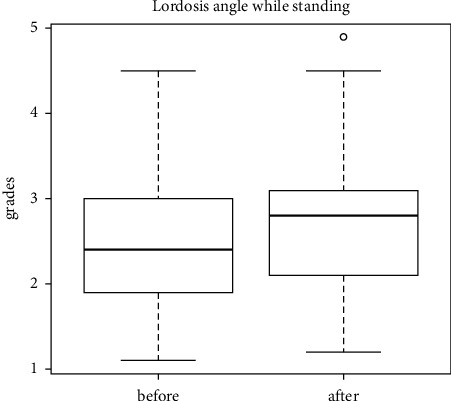
Distribution of the pelvic range of motion relative to the lumbar region (anterior-posterior tilt of the iliac alae) during walking.

**Figure 12:**
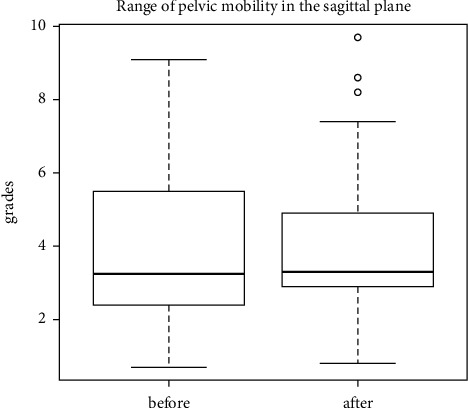
Distribution of the pelvic range of motion relative to the lumbar region (lowering-lifting of the iliac alae) during walking.

**Figure 13:**
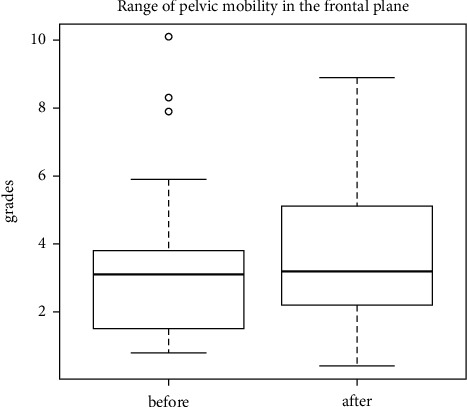
Distribution of the pelvic range of motion relative to the lumbar region during rotation.

The last examination of the study was aimed at assessing pelvic mobility in 3 planes. The distribution of the pelvic range of motion relative to the lumbar segment is as follows: in the sagittal plane (anterior-posterior tilt) (Figure 11), in the frontal plane (lowering-lifting of the iliac alae) (Figure 12), and in the transverse plane (rotation) (Figure 13). No statistically significant differences in the mean levels of deviations were found, which may correlate with the presence of sacroiliac joint locks in 29 out of 30 patients.

## 4. Discussion

The relationship between TMD dysfunction and pain in the other TMJ disorders of the human locomotor system is still an important aspect of scientific investigations. However, cross-sectional studies in postural diagnostics, which is used to assess the relationship between TMD and body posture, have not brought any explicit recommendations in this regard. Scientific reports focus on postural diagnostics in the course of TMD assessment and treatment. The approach to the problems of posture is selective, which is largely due to the complexity and multifaceted character of posture diagnosis and functional assessment of the body.

Saito et al. analyzed the effect of TMD on postural dysfunctions, denying any connection in this regard. The conclusions of these authors were based on the research conducted in a group of 10 patients with displacement of the articular disc in the TMJ and in a control group of 16 individuals with no TMD. The postural examination included photogrammetry, where control points were determined on the body by palpation and inspection. The study showed no difference in the measurement of the trace (longitudinal arches) in patients with TMD and those from the control group. Nonetheless, it was shown that the position of the pelvis, head, and spine significantly differed in the sagittal and frontal plane between the groups. However, given the important biokinematic and structural connections between the pelvis and lower limb, the lack of a relationship between these structures is puzzling. According to the authors, this could have been associated with the use of a plantograph for assessing the feet, the so-called Harris mat [46]. This device can produce a significant measurement error, mainly due to the fact that in order to imprint the footprint by the use of ink, the patient needs to reach a very small diagnostic area, and therefore, his/her position is forced. The study conducted using EMG by Valentino et al. on a group of 10 young patients without stomatognathic system disorders demonstrated that stimulation of the foot increases tension within the masseter and the temporal muscle [47]. Examining a group of 40 women, Kittel-Ries and Berzin noticed that people with TMD showed greater postural asymmetry and pain of the uterine cervix, which was potentially associated with the increased postural stability [48]. Cuccia, who analyzed the relationship between the mandibular position and posture on a much larger group of subjects, showed differences in the arches of the feet between TMD patients and those without TMJ problems. A pedobarograph was used to assess the plantar part of the foot only while standing. This repeatable method of computer foot diagnostics largely eliminated the measurement error resulting from the forced position. However, gait evaluation was omitted in the study. The analysis included the measurements taken in the resting position of the mandible, during teeth clenching and occlusion forced by cotton rollers [49]. Similarly, Souza et al. assessed the relationship between TMD and locomotor system disorders. The tests were repeated at various positions of the mandible, taking into account the TMJ alignment correction. In patients with TMD, the evaluation of the association with the feet included measurements of the distribution of plantar pressure using a pedobarograph. In addition, photogrammetric methods were used to evaluate posture. A significant relationship between the position of the feet and TMD was found [50]. In their research, Walczyńska-Dragon et al. evaluated a group of 60 individuals with TMD for the effects of the stomatognathic system correction. The study included the functioning of the TMJ (mandibular movements and acoustic phenomena), pain, and the range of motion of the cervical spine. The results of the research showed an improvement in the condition of patients in both research areas, which indirectly suggests the need for expanded diagnostics when providing reposition splints [51]. Bonato et al. assessed the relationship between the pain of the TMJ and pain in other distant joints. The study was conducted on a much larger population group (338 people). In the first stage, the clinical evaluation of TMD (Research Diagnostic Criteria for Temporomandibular Disorders) was performed; in the second part of the study, pain in other joints of the body was assessed. The relationship was found between these two features [52]. Studying postural stability, Nota et al. has demonstrated that patients with TMD had statistically significant greater acceleration and surface area of body oscillation in maximal occlusion (intercuspidation) and in the resting position with the eyes open. The study was conducted using a stabilometric platform [53]. Due to the close functional and neurological relationship (the results of foot stimulation examinations), most studies indicate the need to analyze the feet both while standing and walking. Logical inference indicates that these relationships shown in the foot-TMJ system have a huge impact on therapeutic management in broadly understood rehabilitation of the musculoskeletal system. In particular, a close relationship was observed between the treatment of the stomatognathic system and changes in posture and functionality of the body after using the splints. It can be concluded that the application of the methods of posture diagnostic screening seems to be necessary during the dental examination and therapeutic activities. These findings might give rise to issues of competency and logistics, hence the need for standardized and common methods of posture diagnostics to observe global patterns, which include posture assessment in the standing position, body balance, and gait assessment (from the position of the feet and pelvis). These examinations, supplemented with photogrammetric anthropometric methods aimed at examining the entire posture, performed before, during, and after treatment, can be used as complementary methods ensuring the effectiveness of the therapeutic methods. It should be noted that the results of this research showed a discrepancy between patients, with no improvement regularity or the lack of improvement, which was perhaps associated with individual features (for instance weight, gender, age of the patient, systemic diseases). The need for individualized diagnosis of body posture was an important conclusion.

The analysis of acoustic phenomena and pain of the TMJ revealed a direct, positive impact of stabilization of the mandibular head in the articular fossa using a temporary silicone occlusal splint. A significant impact of this supply was demonstrated on mean values of the AI describing the foot arch and its functionality in terms of supination and pronation during walking. The results varied and were directly related to the co-existence of defects of the feet and lower limbs, which is an important argument for further research in this regard. Another global pattern observed during standing and walking was the position of the pelvis in relation to the lumbar region and its mobility when standing and walking in all planes of postural assessment. No significant deviations of comparable variables from standard deviation were found. Noteworthily, however, there was a coexistence of sacroiliac joint locks in most patients (29 patients out of 30). Locks of the sacroiliac joints significantly correlate with TMD. A positive correlation between a degree of acoustic abnormalities and locks of the sacroiliac joints was identified on one side and for the general locks. This paper indicated few aspects of postural diagnostics. The results suggest a relationship between the position of the mandibular head in the TMJ articular fossa and individual posture defects. Despite the lack of an evident direction and repeatability of the changes, they were observed and depended on individual posture defects. Despite our knowledge on postural patterns describing the relationship between the position of the head and pelvis, the results of studies are divergent. Given the abovementioned observations, we should rather focus on the influence of individual determinants, posture defects, and the impact of previous surgeries and treatment. Therefore, an individual and interdisciplinary approach to each patient seems to be important. Based on this information and the results of pilot studies, we can conclude on the relationship between TMD and posture defects. However, research conducted on a larger study group of patients is needed. Pedobarography (EPS R1 pedobarograph) and an integrated sensor for postural diagnostics are used in the tests. Both devices use BIOMECH Studio software, which allows for inexpensive, noninvasive easy posture diagnostics without a need for expert knowledge. The examination time was approximately 10 minutes. We collect information on foot defects, functional disorders, body balance, range of motion, etc. The patient can be examined while standing and during motion, the examination also gives an opportunity to assess the needs of the individual locomotor system. The chief limitation of the study is a lack of a control group, therefore is a potential risk of data interpretation bias. Conclusions on statistical population tendencies will be implemented by a research team in a much larger population group.

## 5. Conclusions

1. Diagnostic and therapeutic management in the course of TMD should be holistic. It is reasonable to use postural diagnostics in the dental therapy of TMD.
2. The observed changes are often varied and largely dependent on individual posture defects, which is an important postulate for further research on a large research group.
3. Pedobarography and computer methods of postural diagnostics are valuable diagnostic tools in TMD therapy.

## Figures and Tables

**Table 1:** Correlation coefficients between the level of TMJ acoustic abnormalities and the sacroiliac joint locks by sides and jointly.

Variables	Correlation coefficient	*p* value
Right acoustics and right locks	0.412	0.024
Left acoustics and left locks	0.489	0.006
General acoustics and the number of locked joints	0.562	0.001

**Table 2:** Differences in mean values of the AI before and after stabilizing the mandible in a new position using a temporary silicone occlusal splint.

	Mean before	SD before	Mean after	SD after	*p* value
Left, during standing	22.72	3.118	23.80	2.642	0.019
Right, during standing	22.01	3.117	23.36	3.152	0.045
Left, during walking	20.25	3.974	17.75	5.477	0.019
Right, during walking	19.34	4.423	20.40	3.927	0.294

## Data Availability

The datasets used to support the findings of this study are available from the corresponding author upon request.
